# Distal posterior inferior cerebellar artery aneurysm with cerebellar arteriovenous malformation treated by open surgery: a case report

**DOI:** 10.1186/s41016-019-0161-z

**Published:** 2019-06-07

**Authors:** Takaya Yasuda, Yoshinori Maki, Ryota Ishibashi, Yoshitaka Kurosaki, Masaki Chin, Sen Yamagata

**Affiliations:** 1 Department of Neurosurgery, Kurashiki Central Hospital, 1-1-1 Miwa Kurashiki, Okayama, Japan; 2 Present Address: Shinko Memorial Hospital, 1-4-47 Wakihamamachi, Chuoku Kobeshi, Hyogo 651-0072 Japan

**Keywords:** Intracranial aneurysm, Arteriovenous malformations, Cerebellum, Surgical procedures, Angiography

## Abstract

**Background:**

The detection of a feeder aneurysm and an arteriovenous malformation (AVM) is relatively rare for the intracranial AVM. The distal posterior inferior cerebellar artery aneurysm (DPICAAn) is reported to coexist or relate with the cerebellar AVM. In previous reports about the treatment of a DPICAAn and a cerebellar AVM, endovascular embolization with the sacrifice of the posterior inferior cerebellar artery (PICA) has often been selected. However, there have been few reports of simultaneous open surgery for coexistent cases of DPICAAn and cerebellar AVM.

**Case description:**

A 67-year-old male presented with a headache. We detected a right DPICAAn in the telovelotonsillar segment and a cerebellar AVM primarily fed by the left superior cerebellar artery (SCA). In addition, the nidus was located medially in the left upper cerebellar hemisphere. Magnetic resonance imaging raised suspicions of asymptomatic past hemorrhage in the cerebellar AVM. The left PICA was agenesis, and the right PICA perfused the bilateral inferior cerebellar hemispheres; thus, the right PICA could not be sacrificed. We selected open surgery to prevent any hemorrhagic event from the DPICAAn and the cerebellar AVM. The cerebellar AVM was completely removed, and the DPICAAn was successfully clipped in a single-session open surgery.

**Conclusions:**

Open surgery can be considered for DPICAAn and cerebellar AVM. The anatomical location of the DPICAAn and AVM contributed to the success of a single-session open surgery.

## Background

The arteriovenous malformation (AVM)-related feeder aneurysm is a relatively rare entity that can be detected in 9% of intracranial AVM cases [1, 2]. Distal posterior inferior cerebellar artery aneurysm (DPICAAn) are reported to coexist or relate with cerebellar AVM [3–5], but DPICAAn comprises < 1% of all intracranial aneurysms. Hence, the coexistence of DPICAAn and cerebellar AVM is particularly rare, and the treatment strategy for cases in which these two entities coexist is debatable. Regarding the treatment of the DPICAAn and the cerebellar AVM, endovascular embolization with or without open surgery is often selected [6]. Here, we report a unique case of a DPICAAn with a cerebellar AVM treated successfully with open surgery, and describe the indications for open surgery for DPICAAn with cerebellar AVM, focusing on the anatomical features of the posterior inferior cerebellar artery (PICA).

## Case presentation

**Fig. 1:**
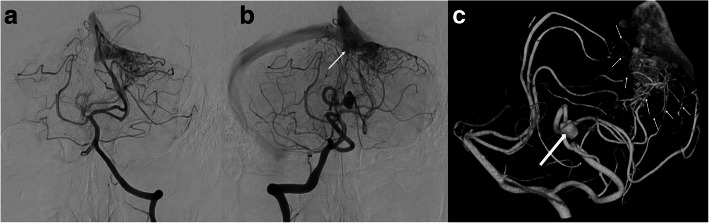
Preoperative angiography. **a** Left vertebral artery angiography (VAG). The left PICA is agenesis (frontal view). **b** Right VAG (frontal view) The tentorial sinus is shown as the drainage route (arrow). The right PICA perfuses bilateral inferior cerebellar hemispheres. **c** Three-dimensional rotational angiography (3D-RA, lateral view) shows the distal posterior inferior cerebellar artery aneurysm(DPICAAn) located at the telovelotonsillar segment of the right PICA with a bleb (large arrow), with neck 3.7 mm and the maximum diameter 7.3 mm; in addition, the AVM feeders are from the left SCA and the right PICA (small arrows). The nidus of AVM is about 18 mm

**Fig. 2:**
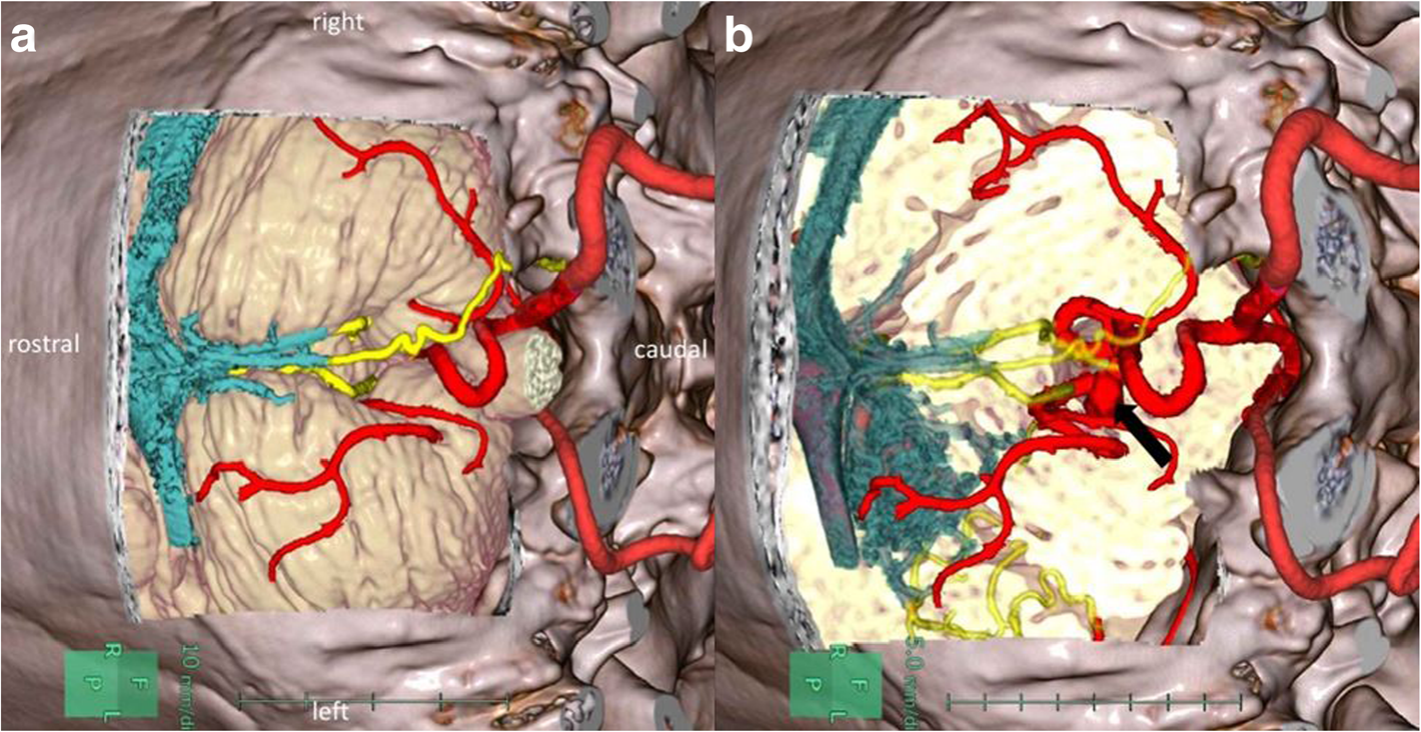
Reconstructed three-dimensional computed tomography angiography and venography. **a**, **b** Three-dimensional reconstructed images, showing the vertebral artery and PICA (red), distal posterior inferior cerebellar artery aneurysm(DPICAAn) (arrow), one feeder from the right PICA of the AVM (yellow), and nidus–drainers–transverse sinus (blue)

A 67-year-old male presented with complaints of a headache. The patient was a previous smoker and had a medical history of hypertension and dyslipidemia. Although no neurological deficit was apparent, a stroke event was suspected. Magnetic resonance imaging (MRI) revealed a right unruptured DPICAAn. A lesion, which was suspected to be a nidus of an AVM, was also detected in the left upper cerebellar hemisphere. The lesion in the left cerebellar hemisphere revealed hypointensity on T2-star image, indicating past hemorrhage. We performed digital subtraction angiography (DSA) to examine the DPICAAn and the possible nidus in the left cerebellar hemisphere. The DPICAAn was located in the telovelotonsillar segment of the right PICA. While the left PICA was agenesis, the right PICA perfused the bilateral inferior cerebellar hemisphere (Fig. 1a, b). The lesion in the left upper cerebellar hemisphere was a nidus of the cerebellar AVM fed by the right PICA and the left superior cerebellar artery (SCA), draining to the transverse sinus through the tentorial sinus (Fig. 1a–c). The Spetzler–Martin classification was grade 1. Reconstructed three-dimensional computed tomography angiography and venography (CTAV) revealed that the nidus was located medially on the tentorial surface of the cerebellum, and the DPICAAn was located beneath the left biventral lobule near the fourth ventricle (Fig. 2a, b). Surgical treatment for the DPICAAn and the cerebellar AVM in the left cerebellar hemisphere was planned to avoid a hemorrhagic event.

### Operation

**Fig. 3:**
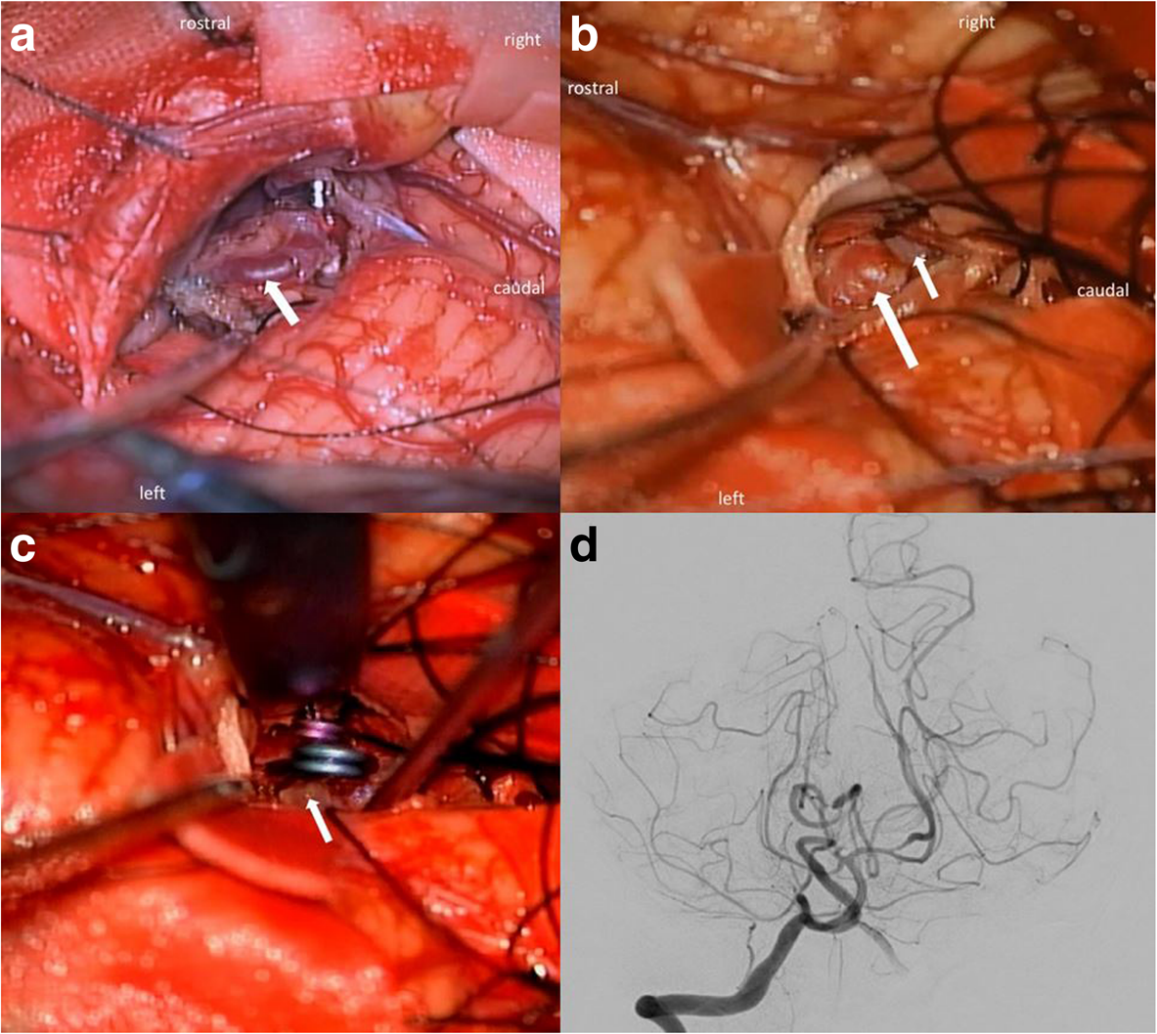
Intraoperative and postoperative images. **a** The nidus and dilated drainers in the left upper cerebellar hemisphere are shown (arrow). **b** The distal posterior inferior cerebellar artery aneurysm(DPICAAn) is shown (large arrow). The proximal part of the right PICA is also indicated (small arrow). **c** The operative view after neck clipping of the DPICAAn (arrow). **d** The postoperative digital subtraction angiography shows the disappearance of the aneurysm and the cerebellar AVM

The patient was placed in the prone position. We selected a linear midline skin incision and midline suboccipital approach. The left occipital artery (OA) was harvested when an OA–PICA bypass was required. After craniotomy, the dura of the cerebellar convexity was cut and retracted superiorly. With caudal retraction of the cerebellum, the nidus was easily detected. We coagulated and cut the feeders from the SCA and the PICA. Next, the nidus was removed after the drainers of the tentorial sinus were cut (Fig. 3a). Next, we opened the left uvulotonsillar space and exposed the right PICA from the cortical segment to the telovelotonsillar segment. In addition, the DPICAAn was exposed (Fig. 3b). We successfully performed neck clipping of the DPICAAn with 2 clips (Fig. 3c).

### Postoperative clinical course

DSA performed 11 days postoperatively revealed the clipping of the DPICAAn and the disappearance of the cerebellar AVM (Fig. 3d). After 18 days of the operation, the patient was discharged from the hospital. No recurrence of the DPICAAn and cerebellar AVM was confirmed with MRI performed 1 year after surgery.

## Discussion

This report presents a case of a DPICAAn and a cerebellar AVM, both treated with single-session open surgery.

A study reported the annual bleeding risk of an aneurysm with an AVM to be 7% [6]. SAH with poor morbidity and mortality can occur in 84% of patients with coexistence of the DPICAAn and cerebellar AVM [5, 7]. The management of unruptured AVM remains debatable after a randomized trial of unruptured brain arteriovenous malformations [8]. In our case, the cerebellar AVM showed possible past asymptomatic bleeding on MRI. Moreover, the DPICAAn comprised a bleb; therefore, we believed that the risk of hemorrhagic events could be high. Although we were unable to conclude whether the episode of headache in the present case was related to the AVM or the aneurysm, we considered that the surgical intervention for this patient was appropriate to avoid any hemorrhagic event.

To date, the surgical strategy of the AVM with feeder aneurysm remains disputable. A report supported the preceding treatment of the feeder aneurysm because the change of hemodynamic stress could lead to aneurysmal rupture when the AVM was first removed [9]. Regression of flow-related feeder aneurysms after the complete obliteration of the nidus by surgical intervention for only AVM has also been reported [10, 11]. In the present case, the right PICA was dominant, which was a normal variation of the PICA and was reported to have the aneurysm at a higher rate than normal PICA [12–14] regardless of a coexistent AVM. The DPICAAn in our case could have arisen from the anatomical feature of the dominant PICA, and not from the feeder aneurysm of AVM. Thus, an intervention for only the AVM may not have resolved the DPICAAn. Next, we decided to operate the AVM and DPICAAn in a single-session open surgery in case of operative bleeding. The DPICAAn was located deeply in the operative field, whereas the AVM was located on the surface of the cerebellum. Therefore, to avoid unnecessary hemorrhage and to safely expose the DPICAAn, we first excised the AVM on the cerebellar surface. Gamma knife could also be a treatment option [5]. However, this technique has some disadvantages, including recurrence of the AVM and hemorrhagic events prior to the disappearance of AVM. Moreover, the gamma knife cannot resolve DPICAAn, and the risk of aneurysm rupture remains. Hence, we did not select gamma knife surgery for our patient.

Surgical approaches for the PICA aneurysms can vary based on the aneurysmal location of the PICA segment, ruptured status, and necessity of posterior fossa decompression. The PICA is anatomically divided into five segments: anterior medullary, lateral medullary, tonsillomedullary, telovelotonsillar, and cortical branches [15]. An aneurysm located in the telovelotonsillar segment, like our case, is categorized as the distal aneurysm [16]. The far-lateral approach is a standardized approach, especially for the proximal PICA aneurysm [17], and the midline suboccipital approach could be a good option for the PICA aneurysm distal to the tonsillomedullary segment [18]. Cerebellar AVMs are categorized into the following five subtypes: suboccipital, vermian, tonsillar, tentorial, and petrosal [19]. In our case, the nidus was located on the medial cerebellar surface and belonged to the suboccipital type. Suboccipital AVMs are supplied by distal cortical branches from the SCA superiorly, PICA inferiorly, and AICA laterally [19]. The vascular supply to the nidus in our case was compatible with typical suboccipital AVMs. For suboccipital AVMs, the surgical approach is ascertained with the location of the nidus on the cerebellar surface [19]. Although the lateral suboccipital approach is the most common, the medial lesion, like our case, can be well exposed by the midline suboccipital approach. Hence, the DPICAAn and the cerebellar AVM in our case were successfully approached by the midline suboccipital approach. Furthermore, the dorsal projection and narrow neck of the DPICAAn, which were confirmed preoperatively on reconstructed three-dimensional CTAV, were also considered suitable for neck clipping. In general, harvesting the OA for the bypass procedure should be considered when the neck clipping of the DPICAAn is difficult.

A study reported endovascular embolization of the AVM combined with parent artery occlusion or microsurgical clipping of the DPICAAn associated with the AVM [5]. As ischemic complications with parent artery occlusion are reported to be 17–40% [4], parent artery occlusion for the DPICAAn seems unsuitable for cases in which sacrifice of the single-dominant PICA such as in our case, can result in fatal cerebellar edema. Parent artery occlusion of the PICA can result in spinal cord ischemia as the posterior spinal arteries originate from PICA in 20–50% of cases [20].

Moreover, the primary feeder of the cerebellar AVM in our case was the left SCA. As interventional embolization of the cerebellar AVM could not be attained only with the sacrifice of the PICA, a single-session open surgery for the cerebellar AVM and DPICAAn was suitable in our case.

## Conclusions

This report presents a case of a feeder aneurysm in the distal PICA and a cerebellar AVM successfully treated by open surgery. In the event that the dominant PICA feeding to the cerebellar AVM perfuses the bilateral cerebellar hemispheres, as in our case, a single-session open surgery could be suitable option. The anatomical location of the DPICAAn and the nidus of the cerebellar AVM contributed to the decision of opting for single-session open surgery.

## Abbreviations

AVM: Arteriovenous malformation
CT: Computed tomography
CTAV: Computed tomography angiography and venography
DPICAAn: Distal posterior inferior cerebellar artery aneurysm
DSA: Digital subtraction angiography
MRI: Magnetic resonance imaging
OA: Occipital artery
PICA: Posterior inferior cerebellar artery
SAH: Subarachnoid hemorrhage
SCA: Superior cerebellar artery
